# Dirty and 40 days in the wilderness: Eliciting childbirth and postnatal cultural practices and beliefs in Nepal

**DOI:** 10.1186/s12884-016-0938-4

**Published:** 2016-07-05

**Authors:** Sheetal Sharma, Edwin van Teijlingen, Vanora Hundley, Catherine Angell, Padam Simkhada

**Affiliations:** KenBerry Solutions Ltd., Nairobi, Kenya; Faculty of Health and Social Sciences, Bournemouth University, Bournemouth, UK; Centre for Public Health, Liverpool John Moores University, Liverpool, UK

**Keywords:** Traditional practices, Nepal, Postnatal period, Women, Babies newborns postnatal care

## Abstract

**Background:**

Pregnancy and childbirth are socio-cultural events that carry varying meanings across different societies and cultures. These are often translated into social expectations of what a particular society expects women to do (or not to do) during pregnancy, birth and/or the postnatal period. This paper reports a study exploring beliefs around childbirth in Nepal, a low-income country with a largely Hindu population. The paper then sets these findings in the context of the wider global literature around issues such as periods where women are viewed as polluted (or dirty even) after childbirth.

**Methods:**

A qualitative study comprising five in-depth face-to-face interviews and 14 focus group discussions with mainly women, but also men and health service providers. The qualitative findings in Nepal were compared and contrasted with the literature on practices and cultural beliefs related to the pregnancy and childbirth period across the globe and at different times in history.

**Results:**

The themes that emerged from the analysis included: (a) cord cutting & placenta rituals; (b) rest & seclusion; (c) purification, naming & weaning ceremonies and (d) nutrition and breastfeeding. Physiological changes in mother and baby may underpin the various beliefs, ritual and practices in the postnatal period. These practices often mean women do not access postnatal health services.

**Conclusions:**

The cultural practices, taboos and beliefs during pregnancy and around childbirth found in Nepal largely resonate with those reported across the globe. This paper stresses that local people’s beliefs and practices offer both opportunities and barriers to health service providers.

Maternity care providers need to be aware of local values, beliefs and traditions to anticipate and meet the needs of women, gain their trust and work with them.

## Background

**Fig. 1:**
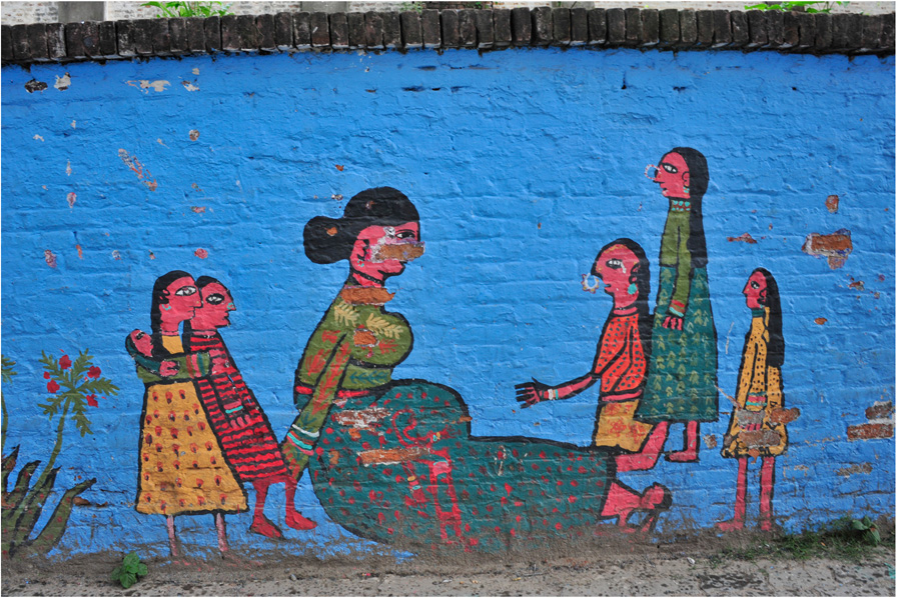
Photograph of a mural of a pregnant woman in Nepal

Pregnancy and childbirth encompass many physiological changes which social and cultural norms influence (Fig. 1). Every society has cultural practices, beliefs, superstitions or taboos concerning pregnancy and childbirth. These can translate into restrictions governed by the family, for instance what women can eat, with many cultures making distinctions between ‘hot’ and ‘cold’ foods, a distinction not necessarily related to temperature or how spicy a particular food is [1, 2]. Moreover, foods are deemed inappropriate (taboo) for consumption in pregnancy or during lactation; in some instances the new mother is perceived as not being hungry and is therefore not fed immediately after the birth [3]. Other examples include limitations on women’s mobility, such as being prevented from crossing a river during pregnancy, which can restrict access to antenatal care [4]. Therefore, traditions or cultural practices may restrict what new mothers can do. The culture or traditions remain very strong even among relatively highly educated women, as will be discussed in this paper.

In countries like Nepal, socio-cultural practices around childbirth include maternal seclusion after labour and birth. Moreover, cultural beliefs in a community play a vital role in non-utilisation of postnatal care (PNC) [5, 6]. Coverage of PNC in Nepal is inadequate, especially among the poorest women and those living in remote rural areas; a recent study suggested that only 21 % of new mothers receive any PNC whether they birth in a facility or at home [6]. Both the neonatal and infant mortality have declined over the past two decades, levelling off at 33/1,000 for neonatal mortality and 46/1,000 for infant mortality [7], while the maternal mortality is estimated at 258/100,000 [8]. It has been suggested that simply providing a postnatal check-up on the first day before women are discharged from the health facility could prevent up to 38.7 % of maternal deaths during that period [4].

This paper explores social and cultural practices that have health implications in the childbirth and postnatal periods of rural Nepali women; and places it in the context of the global literature on such practices. The study explores the extent to which such practices, especially in low-income countries, are part of the cultural adaptation of becoming a mother and identifies why women might not access services, particularly PNC.

## Methods

### Qualitative approach

This study consisted of primary qualitative research on traditional practices around pregnancy, childbirth and the puerperium in rural Nepal. A qualitative approach was considered appropriate for exploring the views of women and health care providers [9].

The study setting was both PNC clinics in a community hospital and participants’ homes, open fields and the village health post in two villages in rural Nepal (identified as A and B for the purpose of this paper). The new mothers went to these clinics for check-ups and vaccinations. The health service characteristics were similar between village A and B. In village A, there were two health posts and a primary health care centre nearby. In village B, the hospital was a community hospital with maternity services (Basic Emergency Obstetric Care Centre) and there were two health posts. Data collection took place in July 2012.

As qualitative methods are most appropriate for exploring complex phenomena [9], interviews and focus groups (FG) were used to explore behaviour and practice [10, 11]. Face-to-face interviews and FGs were conducted with: (a) women with a recent pregnancy and/or with a child under the age of two; (b) their mothers-in-law; (c) their husbands; and (d) healthcare workers in the area.

The sampling was purposive and potential participants were recruited through a network of health centres and women’s groups. Purposive sampling was conducted in order to obtain a broad view of perspectives from a range of participants that included health workers and health service users of diverse social classes. As various ethnicities exist in Nepal (Gurung, Newari, Tamang, etc.) each with their own practices, any cultural issues raised by participants around childbirth were explored in-depth and the all-female interview team enabled postnatal practices to be probed [12]. The FGs and interviews lasted some 45 min each and were recorded (with permission), translated and transcribed [12]. First, five semi-structured interviews involving eight health workers were conducted in English by the first author (SS), as the participants spoke English, typical of higher caste/educated/health professionals in Nepal [13]. The researcher did not have a dual (clinical) role. The interviews were conducted in offices to explore the responses of the participants and gather more and deeper information by probing their answers. Secondly, fourteen FGs (each with 3-9 participants to keep the group manageable but yield good discussion) were conducted in participants’ homes, open fields or the village health post with the aid of a Nepali translator. The latter was a maternal health researcher, as recommended in a study by Pitchforth and van Teijlingen [10]. The qualitative data were analysed independently by two of the researchers (SS and EvT) using a thematic approach. They then compared and contrasted the findings to minimise bias and to ensure the reliability of the data [13]*.*

The Nepal Health Research Council granted ethical approval for the qualitative study (Reg. No. 37/2011 on 1/08/2011). Consent was obtained from each individual participant, and participants were assured that they were able to withdraw, if they so wanted.

### Literature review methods

The literature review on global PNC provision and utilisation was undertaken alongside the qualitative data collection to offer suggestions for areas to explore during or after the interviews/focus groups. We searched the following electronic databases: PubMed (or Ovid MEDLINE), EMBASE, Cochrane Library, PsycINFO, Scopus, Wed of Science, WHO (World Health Organization), CINAHL and Popline. Databases were searched from the start of the database until May 2013 for cultural issues, practices and beliefs. Inclusion criteria were: English language; qualitative and quantitative research; primary studies; all health care settings, including general practice, midwifery, outpatient, clinics, hospitals; all participants; with no time limit. Exclusion criteria were non-English language, papers that did not contain primary research or did not focus on maternity and childbirth. The search terms were ‘cultural practices’, ‘cultural practices AND beliefs related to postpartum/natal period’, and finally ‘40 AND days AND postnatal AND belief’. The term ‘40 days’ was included as a search term as it was mentioned in the interviews and the initial literature search revealed that these 40 days are considered the postpartum period [14].

The qualitative findings are presented first and the captured key study themes are then put into context through an analysis of the global literature.

## Results

The five interviews included eight health workers who were aged between 18 and 37 years old. The interviewees included primary healthcare workers (Table 1). The 14 FG included: 9 groups of women separated into distinct groups of women who had recently given birth and mothers-in-law, aged 17–38; two groups involving men aged 35 to 61 years (some were partners of women who had recently given birth) and two FG with female community health volunteers (FCHV) and one FG with maternal and child health workers (MCHW) aged 32 to 52 (Table 2). Nearly all participants were Hindu. All of the men and two-thirds of the women were literate, at least to primary school level. All 70 participants were married, and many were initially reticent to speak of ‘traditional practices’ for fear of being judged, however as the interview went on they became more confident and open in their disclosures.

**Table 1:** Characteristics of interviewed health workers

Health worker interviews	Age	Village
Hospital staff, Primary Health Nurses (2)	25 and 28	B
Sub-Health Post In-charge, Community Medical Assistant	40	B
Health promoters, Auxiliary Nurse Midwives (2)	30 and 40	B
Maternal & Child Health worker (Outreach clinic)	36	A
Primary Health Nurses/Maternal & Child Health worker (2)	42 and 52	A

**Table 2:** Characteristics of focus groups

Group Interviewed	Age	Village
Mothers with children ≤24 months	25–35	B
Mothers with children ≤24 months	21–28	B
Mothers with children ≤24 months	17–23	B
Mothers with children ≤24 months	26–48	B
Mother-in-law groups	37–55	B
Mother-in-law groups	40–62	B
Female Community Health Volunteer	26–48	B
Maternal & Child Health workers (MCH)	24 and 31	B
Extended family (Husband/Father-in-law) Focus Group Discussions	29–47	B
Female Community Health Volunteer	32–36	A
Mother-in-law groups	55–60	A
Mothers with children ≤24 months	22–28	A
Mothers with children ≤24 months	28–34	A
Extended family groups (Husband/Father-in-law) Group Interview	46–66	A

The key findings will be presented within four themes that permeated from a thematic analysis of the research, starting with: (a) cord cutting & placenta rituals; (b) rest & seclusion; (c) purification, naming & weaning ceremonies and (d) nutrition & breastfeeding. Under each heading the findings from the qualitative interviews are presented, followed by the findings of the literature analysis to put the former into context.

- **Cord cutting & placenta rituals**

Participants discussed how the cord was cut, and how the placenta was expelled and then buried. No participant mentioned eating the placenta. Cord-cutting practices could involve a variety of substances and diverse types of tools to cut the cord, for example one health worker said:

*(Mothers) say to us they have used sickle to cut the cord or razor blade…and then apply sometimes antiseptic, cooking oil, ghee (butter), toothpaste, ash or nothing to the umbilical cord.***Health promoter/Auxiliary Nurse Midwife, Interview**

A few postnatal Tamang women mentioned the cutting of the umbilical cord at home:

*The cord was cut by ‘hasiya’ (scythe)… (and then) cleaned with plain water*. **Tamang mother, FG**

Gurung women commonly bury the placenta at the foot of a tree, while the Newari and Tamang bury it at a junction or under the road. For example, a Tamang mother referred to burying the placenta.

*Then placenta was buried in the road where there is a junction (laughing…),***Tamang mother, FG**

It is believed that the child’s future health is linked to the method of disposal of the placenta:

*The placenta here is buried. It should not be eaten by a crow and should not be eaten by insects. If it happens, it won’t be good for child, they will be sick.***Tamang mother, FG**

There are “low-resource” interventions (i.e. practices) used to expel a retained placenta:

*If placenta is not expelled, a piece of cloth is inserted/packed in woman’s mouth so that she has nausea. That helps to expel the placenta.***Tamang mother, FG**

b) **Rest & seclusion**

Generally, Nepalese women rest after labour and delivery, with duration of rest differing between the various castes. Dates of significance in Nepal include the date when the new mother can leave her in-laws’ home (after 30 days) to go to her parents’ home for a period of rest that lasts from a few days up to a month. At this time her nutrition becomes a priority. One Tamang respondent describes this period:

*… Then the postnatal mother is allowed to go in kitchen on the 9th day. The postnatal mother is sent to her mother’s home at around 10-15 days and she can stay in mother’s home as she wishes, sometimes up to a month.***Tamang woman, FG**

In this time a massage is given to both baby and mother, it typically involves mustard oil to relax the muscles and help the child grow with the smoothing of joints. Nepalese women have a postnatal caregiver who comes to massage the abdomen. In her maternal home, the new mother is cared for and is fed a specific diet of lentils and spices, such as cumin, believed to stimulate her breast milk production.

*…from this day (nwaran* or baptism*) onwards, mother and baby are given mustard oil massage and stay in the sun (laughing).***Brahmin woman, FG**

Most women reported that, unlike in the past (in the last decade) where they would return to household duties soon after birth, they were now allowed to rest in their in-laws’ home. Perhaps, the underlying issue is that there existed a lack of awareness of the physiology of pregnancy and childbirth; that in the postnatal period women need ‘time to heal’ and were therefore ‘isolated’. Isolating pregnant women in the shed was also mentioned during the men’s FG, typically in a cowshed. In Nepali the word for such a shed is ‘chhaupadi’, which is also the word for this excluding practice:

*There is not such an influence or effect in this village, but in some place there is still the practice of isolating women during menstruation/pregnancy (chhaupadi), sometimes the women have to stay in the stable (shed) also. In my thinking in this place (cough) there are not so bad practices. Everyone is doing equity behaviour (treating women equally to men).***Tamang and Newari Men FG**

c) **Purification, naming & weaning ceremonies**

The qualitative data indicate that in rural areas of Nepal, a purification ceremony is performed as birth is believed to be unclean. The naming and purification ceremony *nwaran* involves a day of cleaning the home, bathing the mother and baby, and choosing a name. The ceremony takes place anywhere between the 3^rd^ and 12^th^ day after birth. A priest performs the ‘baptism’ ceremony of “*nwaran*” at home, which includes the horoscope from the child’s birth details, and the mother and child are ‘purified’ (from their ‘past birth’, in the religious sense).

In the days before the *nwaran* ceremony, the new mother cannot go out. She should not visit the (Hindu) temple and no one will touch her or take the child directly from her, as a vaginal birth is deemed ‘dirty’. The mother-in-law will use her old sari or old clothes to pick up the child. In the past women would warm/cleanse their hands over the fire before holding the baby. Finally, fire is also used to burn the woven mat on which the *Bahun* women lie during and after childbirth.

Thus, birth is considered ‘dirty’ and the *nwaran* ceremony comprises the cleansing of the mother and house as well as naming of the child.

*Delivery is considered dirty and untouchable. Usually, nwaran … here takes place on 3rd day of delivery. Some (families) invite the lama (local Tamang priest) to pray and purify home and to name the newly born baby.***Tamang woman FG**

After the *nwaran* ceremony the new mother is included back into the household activities. The sleeping child is placed in a sari, as the used cloth is believed to offer protection from the ‘evil eye’:

*The evil eye is averted when you have the baby sleep in the mother-in-law’s sari; even mothers who are teachers (or who are educated) follow these practices.***Brahmin new mother and schoolteacher, Interview**

Until they are ‘cleansed’, women have other restrictions imposed on them, including not being allowed near a deity’s statue or a temple (Nepal is a strongly Hindu country) during late pregnancy:

*She (mother) is not allowed to worship God from around the 6th month of pregnancy until nwaran****.*Bahun and Chhetri mothers FG**

Furthermore, a secret *nwaran* name based on their ‘*rashi’* (astrological sign) is given to the baby in Nepal. This rashi is astrological and dependent on the time of day of the baby’s birth.

*Nwaran celebrated on 9th day for girl and 11th day for boy, which at the same time they do the purification ceremony. The name is given based on time, day and date of the birth. The Jyotishi (astrologer) is responsible to extract the name for the baby.***High caste (Brahmin) health worker, Interview**

The new mother in Nepal also has dietary restrictions.

*Until the nwaran purification day, (the) a mother is not given salt, green vegetables, and is not exposed to the sun. Nwaran is done on 9*^*th*^*day of delivery both in Bahun and Chhetri communities.***Local hospital staff, Interview**

Among some Tamang there are two stages of purification *chokyaune* usually on 7^th^ day of delivery, and *nwaran* usually on the 9^th^ day and on that day the mother and baby are taken outside for the first time. Then they are considered ‘clean’ and can be ‘touched’ by others.

*We cannot go to the kitchen, we stay (in) one corner of the ground floor; we cannot go up the stairs (laughing).***Tamang mothers FG**

The laughter seemed to be more about the perceived oddness of the situation rather than embarrassment.

The father is part of the cleansing ritual *nwaran*, after the child’s mother is allowed outside the house.

*Then only we are taken out of the home in sun. Usually the child’s father does hom (special worshiping) on the third day also.***Tamang mothers FG**

These ceremonies vary between different ethnic groups. *Nwaran* takes place on the 3^rd^ day among in Tamangs, the 7^th^ day among the Bahun and Chhetri; and in Newar communities between the 9^th^ and 12^th^ day. It seems the higher the caste the later the ceremony. Similar to the rice ‘weaning’ ceremony *pasni*, *nwaran* takes place later for boys than girls.

*Keep her (the mother) warm, give hot food, oil massage, and keep in the sun, burn lamp on 6*^*th*^*day and rice feeding on 5*^*th*^*month for daughter and on 6*^*th*^*month for son*. **Tamang and Newar FG**

The rituals carry on until the 10^th^ day:

*On the 10*^*th*^*day lanterns surround the baby’s mother. Nwaran is done usually on 12*^*th*^*day for daughter and 5*^*th*^*or 7*^*th*^*day for a son in the Newar community. Baby and mother are given a bath, and then the priest comes home and organizes the puja (prayers). Then, (they are) considered purified. Afterwards, mother and baby (are exposed to the) sun every day*. **Newar hospital staff, Interview**

Alongside *nwaran*, cow urine is used to purify the walls of the house. Family members of the newly born baby consume a drop of cow urine and cow dung is used with red soil to clean the house; as the cow is considered the national and holy symbol of Nepal.

The weaning *Pasni* ceremony occurs at 5 months for girls and at 6 months for boys. In this ceremony solid silver anklets called *khalis*, carved with dragons at both the ends, are given to keep the bad omens away from baby, and they are believed to help the baby’s legs grow stronger. Gifts are given to the child and the mother may observe a fast. The fasting then coincides with weaning of the baby and the introduction of solid food.

d) **Nutrition & breastfeeding**

Food practices mentioned by women in the interviews included special attention given to nutritious food and the use of aryuvedic medicine. The new mothers are given a special diet in the postnatal period like “*kwati*’, a special soup prepared from various beans with some meat. Several Tamang women said that “*dahi-chyura*” (curd and beaten rice accompanied by meat curry) is postnatal food. Mothers are also given “*gudpakh*”, a special sweet in the form of a cake, rich in calories, made from flour, clarified butter, cashew nuts and coconut.

*Mothers take gudpakh; mother becomes strong during pregnancy time. They prepare it at home. Those who visit the mother, they get it from outside. At home they prepare with milk… till baby is 2 years old we put a chain around their neck made of the umbilical cord.***Newari mothers-in-law FG**

Additionally, *janma ghuti* (a commercial aryuvedic medicine for digestion), *balmrita* (herbal aryuvedic medicine) and *jaiphal* (nutmeg) and herbs and spices are given to the newborn baby. Traditional healers use these as medicine.

*Some of the aryuvedic foods include quati quati or ghuti aushodi (a soup of mixed pulses); in it is supari (crushed betel-nut), jaiphal, pepper and cashew nut…So that baby’s heart become strong and even for strong bones.***Newari mothers-in-law FG**

## Discussion

The qualitative research conducted in Nepal highlighted that (a) birth was perceived as ‘polluting’; (b) postnatal women were perceived as being ‘polluted’; and therefore isolated and (c) cleansing rituals were required for mothers after the resting/isolation period. Consent was obtained from each participant, this was particularly important within a culture where most women have to ask their husbands. Although, the husband’s permission is needed, during the interviews and focus groups the discussions were ‘organic’, i.e., women openly spoke of their beliefs and practices.

There was a major overlap with existing literature, with the themes of the qualitative study (cord cutting and placenta, purification, naming and weaning ceremonies, rest and seclusion, nutrition and breastfeeding) echoing what researchers had found in other countries. This sets the study in a wider global perspective.

- Cord cutting & placenta rituals;

In Nepal, it is considered lucky to cut the umbilical cord on a coin [15]. The treatment of umbilical cords is very ritualistic, and various household tools are used to cut and tie the cord. The qualitative research suggests that in rural areas the cord is often cut with a sickle or an unsterilized knife, a practice noted in similar communities in Bangladesh [3, 16].

Poor cord hygiene is a common issue in many low-income countries and particularly in births taking place outside of health facilities. For instance, in India the tool used is related to the trade among the caste; for example the use of a scythe by farmers, however tetanus is reported in new-borns [17, 18]. In Bangladesh, the cord is only cut after the placenta is delivered; the ‘cord cutter’ remains ‘unholy’ and cannot go for prayer for 41 days. The mother is already considered unclean due to having recently given birth, so she can cut the cord, as can a child that has not begun to pray as (s)he is also considered to be unclean [3].

In Nepal the placenta is generally buried, to protect the baby. If the placenta is retained the practice is to try to make the woman vomit to help expel it; while in other low-income countries accounts exist of massaging and sitting on the abdomen [19]. The practice in Mayan Yucatan is to treat a retained placenta with abdominal massage, applying hot water and alcohol, and then covering the woman with blankets [20]. In Malaysia, the midwife massages the mother’s abdomen after the birth to facilitate the expulsion of the placenta [21].

All of these are low-cost, but not necessarily low-risk, interventions to address the problem of a retained placenta. However, the placenta’s “low-resource” practices mentioned are not without risk, indeed as the literature shows sepsis remains the major cause of neonatal mortality in Nepal and the second leading cause of maternal mortality. WHO recommendations for achieving a clean birth include a clean surface for delivery, clean hands of the birth attendant, clean cutting of the umbilical cord, clean perineum, clean cord tying, and clean cord care, since use of household tool and substances may lead to sepsis [22, 23]. It has been estimated that these clean birth practices can avert 20–30 % of newborn deaths due to sepsis and tetanus [24].

In the literature, many cultures link the baby’s demeanour and future with the placenta. Placenta, the Latin word for cake, is referred to in France as a baked good; the ‘other’ bun in the oven [17]. Furthermore, a placental recipe from 1983 published in the magazine *Mothering* mentions the oxytocin contained within the placenta might prevent postpartum haemorrhage; placentaphagy benefits are known [17, 25]. However, we found no evidence of this in our study in Nepal.

There are also rituals associated with placental burial. For example, placentae are buried at a junction in Mexico, similar to the Newari community in Nepal [17]. One possible explanation can be identified from Indian, Semitic myths; old Jewish texts tell pregnant women not to stand alone at the crossroads as they may “*see the foetus taken away by evil powers*”. It seems a crossroad is the place where spirits dwell [26]. Perhaps burying the placenta at a crossroad diverts evil spirits away from the new baby towards the ‘useless’/less important placenta. Similar to Nepal, in Lao the placenta is considered a dirty object to be buried and a fire is lit over the buried area in order to prevent spirits and animals from reaching it. If any part of the woman touches the placenta, it is believed that the lochia might dry up, causing harm to her baby and even neonatal death [27]. Lao and Burmese ethnic women still practise traditional childbirth rituals during birth preparedness, umbilical cord cutting, where they ‘roast’ or provide heat to mothers to stimulate healing [2, 27]; a practice also seen in traditional medicine in Laos [28].

(b) Resting and Seclusion

From the data it seems that women were housebound for a number of days after the birth and the length of this period of seclusion varied by caste or ethnic group. This is a phenomenon found across the globe, including in high-income countries in the recent past. The length of time a woman is secluded or rested varied across different countries and the principles underpinning this isolation (to heal vs. being unclean) also seem to differ greatly. After the period of seclusion there is often a ceremony to purify women to publically accept them back into daily life. The literature supports the concept of a resting – a lengthy lie-in or lying-in period, a period of seclusion, as women need to rest in order to heal, yet it may mean that they are neglected. In Greece, birth customs include women and babies resting and being isolated for 40 days after birth, a period that is still observed [29]. The 40-day period is called the lochial period, from ‘lochia’ the normal vaginal discharge of cell debris and blood after birth. The Bible says “40 days” for the vaginal discharge resulting from involution and can also be described as the red lochia, lasting 4–6 weeks [29]. The lochial period is a time when the “woman can be cherished and pampered without feeling inadequate or shamed”, noted Mead and Wolfenstein, some 60 years ago [30]. As mentioned in the interviews, in remote rural parts of Nepal women are isolated made to birth in the cowshed ‘chhaupadi’; women menstruating or in labour are thought to be ritually polluted and must be kept at a distance from the family in these sheds [31]. Women in Zaire and India are also secluded in a hut [32]. For Muslims the period of postnatal seclusion traditionally lasts 40 days. The religious rituals are performed on the 40^th^ day and these include shaving the child’s head, as a vaginal birth is considered unclean. This act permits, what is considered, the growth of ‘new’ and ‘clean’ hair [33]. This ‘seclusion’ around the time of birth also occurs in Burma and in Turkey where it is believed that postnatal women are more vulnerable to evil forces and “the grave of women who have just given birth is open for 40 days”: postnatal women are at risk and can easily die in 40 days and in that period, mother and baby are not left alone, lactating women do not go out, they and their children are not bathed [2, 34]. This is in contrast to women in Nepal; where they are left alone. Purdah (female seclusion) is observed in Bangladesh lasting 5 to 9 days and there exist dietary restrictions that last up to 6 months [3]. Similarly, among the Negev Bedouin in Israel, a 40-day postnatal period includes seclusion (homestay), followed by congratulatory visiting, the reciprocal exchange of gifts and money, and observance of a special diet [35].

A number of cultures have beliefs, taboos and behaviours relating to women and newborns in the postnatal period, a period lasting up to 40 days. Among Mayans the period lasted 20 days and Japanese mothers remained in a birth chamber for 3 weeks [17]. In Chinese the postnatal period of rest is called the ‘sitting month’ or ‘doing the month’ and lasts for 30 or 40 days. This exists, according to Chinese traditional medicine, as postpartum women are considered to be in a ‘weak’ state, and the practice is still observed with primiparous women [1]. Keeping mothers together with their babies is medically important but also culturally: in southwest Nepal new mothers stay with their babies continuously for 6 days [36]. Higginbottom refers to a 40-day period after the birth in which particular foods are eaten [37]. Cassidy also refers to “the upsitting” where bed linens would be changed and on the 10th day the mother was allowed to perform housework, and that hard labour ought to be avoided in the weeks after birth for the risk of uterine prolapse [17]. Burmese women also observed rest in the postpartum period [2]. The 40-day period has often been put into practice as the ‘quarantine’ period for women, a period of rest and purification [17]. The word “quarantine” originates from the Venetian dialect *quaranta giorni*, meaning ‘forty days’ for the length of isolation of ships for detection of plague symptoms [38]. This separation of infected people was used to prevent the spread of disease, and is recorded as far back as the Old Testament [39]. Culturally and historically, birthing women are considered ‘unclean’ [2, 19]. In many cultures postnatal women are believed to be dirty and weak [16, 40, 41]. Moreover, the pollution of birth is detailed; for example in Nepal, Maori (Aetoroa/New Zealand), Japan, China, Inuits in Canada, Turkey, and Bangladesh [1, 3, 16, 17, 34, 40].

Evidence of isolating practices can also be seen in western countries. In Europe in the recent past women were considered ‘polluted’ and dangerous to men, so new mothers were not allowed to prepare or cook food for 40 days [17]. The immediate period after childbirth is referred to historically as the ‘lying-in period’ in English and “Wochenbett” in German or “week bed”. Browne and Browne refer to the lying-in period as 8–10 days after labour and birth; similar to the time it takes for the stump of the umbilical cord to fall off naturally [42].

Historically women in the British Isles were unclean after birth [43]. Purification as a ritual is likely to have at least some physical foundation, such as notion of infection control in modern medicine. The 40-day period presents vulnerability in mother and child which can be targeted in this time; as a frequently described postpartum problem is infection [3].

In the USA, self-help books on childbirth inform new mothers and their partners that the postnatal period lasts 6 weeks [44]. Six weeks is, of course, a different way of expressing ‘the 40-day period’. The explanation such self-help books give is that “the uterus has returned to a non-pregnant size and bleeding has abated” [44]. Similarly, one of the first UK guides for new mothers recommended that women visit their doctor at 6-weeks postpartum for a range of physiological check-ups (a period of 6–8 weeks for uterus and other pelvic structures to ‘heal’ the puerperium), [45, 46]. Eastman and Russell also suggested that energy demanding activities such as tennis, cycling, jogging and heavy housework/lifting be postponed until the “lochia has ceased” [29]. In the 1960s, Browne and Browne claimed that red lochia lasted 24 days and only after that time should women resume household duties, start going out again or drive a car, whereas the shampooing of hair could be done as desired [42].

Caring or nourishing of women during this period is seen in the literature. In Nepal, women can have a postnatal massage to the abdomen in order to promote blood circulation and therefore healing in the first weeks post-childbirth [36]. Mayan women get “one or more massages” from their midwives 28 days post-partum [47]. In Nepal, our findings were that traditional postnatal care includes baby massage with mustard oil, massaging the mother, and an emphasis on nutrition. In higher castes (Brahmin, Chhetri, Newar and Bahun; in Nepal Tamangs are lower caste) these tasks are performed by a birth assistant, who will stay in the house for a month to wash the child’s clothes and cook for the mother.

Mothers need rest and seclusion, thus there are advantages for new mothers of having a lying-in period with its associated rituals and taboos. The historian Cressy (1993) uses the term postnatal privileges to reflect this positive notion [48]. Women need rest after childbirth but should not be treated as ‘infected’ or ‘dirty’ during their seclusion period; research from China has found that the 40-day seclusion custom can adversely affects women’s mental health with reports of postnatal depression occuring due to the feeling of isolation [49]. Also adversely affecting women’s mental health are folk beliefs or traditional attitudes around stillbirth, which are slightly different in Nepal. This might reflect a lack of research on the impact of stillbirth on maternal mental health. Another concern is the issue of alcohol consumption as mentioned by some ethnic groups in Nepal, although this appeared to be a less common issue globally. Another issue of concern is sexual violence as the prevalence of sexual violence within marriage ranged from 12 to 50 % in Nepal [50]. Lastly, three studies have reported post-partum depression among women in Nepal to be between 4.9 and 12 % [51–53]. However, postpartum depression, it seems, is not discussed with women from low-income countries [54].

(c) Purification, naming & weaning ceremonies

The Hindu caste system and its associated behaviours have an impact on birth customs. Similar to Nepal, in India the naming ceremony takes place on the 10^th^ or 12^th^ day after birth after which the mother is considered ‘clean’ and can carry out normal household chores (e.g., cooking); furthermore male visitors can visit the nursing mother. A weaning ceremony at 6 months (*Annaprassana*) is believed to be necessary for the baby to become more mobile; gifts here too are given to the child and the mother may observe a fast. Glass bangles worn during pregnancy are gifted to the midwives. Mothers in India also return to their parental home for 40 days after the birth. These customs are also practised by the Hindu diaspora and can lead to antenatal and postnatal non-attendance [40]. The literature also demonstrates the religious importance of ritual cleansing. Traditionally the Church of England had a thanksgiving ritual welcoming new mothers back in the church after childbirth, which was also a ritual cleansing ceremonial. The ritual referred to as ‘churching’ lasted well into the twentieth century [43]. Similarly, historically ‘kirking’ was found in the Highlands of Scotland. Associated with the Church of Scotland, it referred to the cleansing ritual to allow the women polluted in childbirth to come back into the kirk (church) [55]. The Greek Orthodox Archdiocese in the U.S.A. states that women may stay home for a period of 6 weeks after giving birth [56]. *The Holy Bible* in Leviticus XII: 2 notes that where the woman

“born a man child: then she shall be unclean seven days; according to the days of the separation for her infirmity shall she be unclean.” *…* “And she shall then continue in the blood of her purifying three and thirty days; she shall touch no hallowed thing, nor come into the sanctuary, until the days of her purifying be fulfilled” (*The Holy Bible*, Leviticus XII:4) [57].

Similarly, Jewish women were allowed back into the temple 33 days after the birth of a son and 66 days after the birth of a daughter [17]. The notion of purification in the 40 days also denotes the temptation of Christ when Jesus was in the wilderness “And when he had fasted forty days forty and forty nights…” (*The Holy Bible*, Matthew, IV: 2) [57]. We must bear in mind that in *The Bible* 40 days may refer to a long period of time rather than exactly 40 days [58].

Ceremonies frequently involve burning as part of the cleansing. In Indochina fire in the postpartum period plays a central role in ritual cleansing. In Lao PDR the confinement period of rest and “lie by the fire” is perceived as positive, the ‘bad blood’ bleeds out as women lie on the floor [28]. Furthermore, women in Laos refer to being ‘roasted’ and that advance preparation of, for example, baby clothes would lead to death of the newborn [27]. Interestingly, allopathic practitioners have now incorporated some of these traditional practices in Laos [28].

(d) Nutrition & breastfeeding

The literature discussed the role of food in the postpartum period. In Bangladesh on the first day after birth, to continue the healing of the birth passage, no food is given, and in the following days meals are nutritionally deficient consisting of rice only, as polluted women are not perceived to be hungry. Burmese women and women in Turkey who adhered to traditions of food restrictions and prescriptions during the postnatal period were traditionally not given any water to drink for 2–3 days after the birth [2, 34].

There are many references in the literature to hot and cold foods [40, 59]; and it is worth noting that hot in one country is not necessarily hot in another [60, 61]. For instance, China has the notion of ‘Qi’ deficiency and blood loss, ‘heat’ or ‘cold’, which may cause health problems like dizziness; thus ‘cold’ foods should be avoided ‘hot’ should be encouraged [1]. This notion of hot and cold also exists in Laos, whilst taboos include not bathing, no hair washing or teeth brushing and staying in bed between 18 h to 2 days [1, 2]. The notion of ‘hot’ and ‘cold’ is not only related to the food, but can also relate to the stage of pregnancy and birth. In Malaysia pregnancy is ‘hot’ [59], in Cantonese China the pregnant mother is ‘cold’ and the foetus ‘hot’ [62], whilst in Vietnam both the mother and foetus change from ‘cold’ in the first trimester to ‘hot’ in the last [41]. The notion of ‘hot’ and ‘cold’ with regards to pregnancy also exists in Laos [28]. In the literature however there was no indication of herbal aryuvedic medicine/food being harmful, suggesting a gap in existing evidence.

Dietary and breastfeeding restrictions exist; some offer women poor diets for a variety of days in Laos [28]. In Nepal, India and elsewhere in South Asia, colostrum is not given until a priest approves it, as it is considered to be pus [3, 17]. This is not unlike seventeenth century England when medical texts recommended against the feeding of colostrum [63]. In common with Nepal, the initial breastfeeding practice in Bangladesh is poor, as colostrum is not given, as it is deemed to be ‘dirty milk’ due to its pus-like appearance. Taboos are also evident in relation to the baby. In Bangladesh in the first 40 days breast milk is given; as is sweet water “*misri pani*”. The latter is thought to have benefits. While, in richer households goat or cow milk is given after 40 days, yet in poorer houses *misri pani* often leads to a high incidence of diarrhoea [3]. The breastfeeding diet is observed for 40 days. In Cairo, infants are breastfed exclusively for the first 40 days after birth [64].

The majority of women in the qualitative interviews reported that they discarded their colostrum, which they felt was inadequate in nutritional value. Whilst this is contrary to the World Health Organization (WHO) recommendation that breastfeeding should commence in the first hour after birth [65]; it is significant because it indicates that women were actively expressing colostrum. This is vital in terms of stimulating their breast-milk supply [66]. This may be why the practice of discarding colostrum as done in South-East Asia is not as detrimental as previously thought [67]. Although, this is less than ideal in terms of the beneficial constituents of colostrum and definitely harmful if other substances are given, such as honey, butter or unclean water. Breastfeeding rates are high in Nepal (although not necessarily exclusively breastfeeding) when compared with UK rates at 6 weeks and 6 months, but not on the first day or two, as is common in South Asia. Some authors have suggested that women in South Asia generally do not breastfeed on the first or second day; but they do stimulate their breasts for the milk supply [3, 33]. Breastfeeding statistics for the UK show reasonably high initiation rates (81 %) but at 6 to 8 weeks the prevalence is down to only 47.2 % [68, 69]. Breastfeeding is associated with reduced risk of infection (colostrum contains elevated concentrations of multiple antimicrobial proteins), prevention of dehydration and hypoglycaemia in babies and reduced risk of breast and ovarian cancer in mothers and increased mother-baby bonding. Breastfeeding has short and long-term health benefits for both baby and mother [70]. Potential long-term health benefits in children include reduced blood pressure, cholesterol concentrations, and obesity [67, 70].

The literature illustrates that alcohol plays an important role in both birth and the postpartum period. In Nepal the Tamang mothers drink *jad* during their pregnancy and post-pregnancy, similar to the ‘god-sips’. The term ‘god-sips’ is thought to have arisen because when a woman went into labour, her ‘gossips’ were sent forth to gather for merriment and to partake in a drink at the labour [71]. Indeed the drinking midwife is mentioned in Shakespeare’s Twelfth Night: “like aqua vitae with a midwife” [72]. In the Tamang community *jad,* an alcohol, is taken during pregnancy and post-pregnancy to celebrate the birth, and Gurung women may drink it to put the child to sleep during breastfeeding as alcohol will certainly pass into the breast-milk [68].

Alcohol and pregnancy are linked culturally, for instance in Africa rum was given to the Akan and Igbo child. Furthermore, the birth was celebrated with alcohol and at the naming ceremony [73]. Drugs passing into milk as cathartics were identified by Greek physicians and Gurung women use it to put the child to sleep [29]. Alcohol is also used in the protection of children; Malaysians bathe children in stout as they believe it protects babies and to help new-borns suffering from jaundice [32, 74].

This article has discussed that there are logical reasons for practices that are linked to the physiology of pregnancy, birth and the postpartum period. The umbilical cord if left alone typically falls off after six to ten days, whilst the lochia heal in about 40 days/6 weeks [75]. The latter ties in with the International Classification of Diseases’ definition of maternal mortality [76]. However, the origin of the 42 days limit is historical, i.e., in the old Anglican Church and the Jewish faith where purified women resumed attending prayers 40 days after childbirth, rather than medical. Clinically, the relevance lies in the first menstrual bleeding in non-lactating women occurring 6 to 8 weeks after parturition. The literature finds that the 42 day limit was not based on a study of the timing of maternally related deaths [14]. Some practices, however, clearly put women at risk; isolation may mean rest but if the woman is alone and suffers a postpartum haemorrhage, this may result in a preventable maternal death. Also unclean tools such as a scythe used to cut the cord may lead to infection, or fasting can lead to malnutrition, and most commonly the discarding colostrum may reduce the protective effects of early breastfeeding.

It is easy to forget that childbirth is a hazard for mother and child in many low-income countries; some traditional practices reduce and others increase the chance of dying. This article highlights that cultural practices exist universally between days 3–10 and 40, and that many of these can be linked to physiology. The timing of these important events means cultural influences play a role in postnatal practices [77]. In society, rituals develop over time to deal with the physiological and social aspects of birth and are internally consistent.

The study and literature within this article have shown that reproductive health is shaped by culture and women’s position may be influenced by social and cultural aspects rather than biological factors: The role and place of women in society is ‘lowered’ in a patriarchal society where historical social norms are maintained. Several studies refer to cultural sensitivity when dealing with women, focusing attention on improving the maternity services rather than on women and their cultural differences [12, 78, 79]. As social cultural practices are passed down from senior females to younger generations, postpartum home visits may play an important role in helping women to change behaviours [1]. Nepalese maternity care should focus on the rural population to be more sustainable and maternity nurses/midwives can use health promotion interactions during home visits [37].

### Strengths and limitations of the study

This is one of the first studies of its kind in Nepal. Women were interviewed individually, which allowed them to speak about the issues anonymously. However, due to accessibility, time and resource constraints men had to be interviewed in groups. This is a limitation as male participants in the focus group stated that the topic was women’s ‘business’ and they felt that they could not comment in any depth. A minority of the interviews were conducted in English, which may have influenced the way Nepali professionals expressed themselves. Most interviews and all focus groups relied on a translator, which also may have affected the data. The translator had a health background and was trained prior to the research and the interviewer spoke Hindi and a few words of Nepali which helped ensure the quality of the data. A limitation to the search strategy is that it did not include ‘stillbirth’ and ‘nutrition’.

### Policy relevance

A gap in knowledge surrounding social cultural conditions may explain the failure of some health policies and programmes to address such issues [80]. Therefore, it is important that, even in the postnatal period, childbearing women feel they can discuss non-health worries that relate to superstition, myths and taboos. Culture and traditions are fraught with ambiguity, especially as many health programmes aim to integrate ‘evolving modernities’ with the influence of globalisation [37]. Furthermore, social cultural practices can affect women’s health status, and therefore a westernised model of care is not advocated, rather informed decisions should be taken regarding locally appropriate illness prevention. In addition, health policymakers and international development advisers need to take social and cultural conditions into consideration to formulate evidence-based policies to reduce morbidity and mortality in mothers and their babies, and reduce gender inequalities [80–82].

### Implication for practice

Understanding childbirth values and beliefs of specific cultural groups can promote culturally appropriate evidence-based care. Cultural postnatal practices can be harmful or ineffective, but changing deep-rooted practices, often with religious origins, is challenging even among educated women. Understanding the social cultural environments should be part of health providers training to change these behaviours or incorporate them into the care. The clearly detrimental behaviour will require culturally sensitive re-educative programmes that create new understandings in both practitioners as well as women of childbearing age and their family and local communities. Some of the interventions should address the physiology of childbirth, which is often poorly understood by rural women and/or those with low education levels. If local people know how their traditional behaviour fits with the physiology of childbirth it might be slightly easier to change some of the undesirable or risky behaviours. This understanding is also of importance when designing culturally appropriate interventions: such as birth kits in low-income countries [83].

Finally, practitioners in high-income countries can learn from those in low-income countries to help provide culturally appropriate care that is accessible. This will be especially beneficial to high-income countries with large ethnic minorities to help avoid discriminatory policy and practices.

## Conclusion

Social cultural practices may prevent women from accessing postnatal care. Although there are physiological explanations that underpin some of the beliefs and practices around, for example, a new mother needing rest for a 40-day period, it is important to stress that not all practices necessarily have a physiological origin. The role and place of women in society probably have a much greater negative effect on postnatal women. Some of these influences are negative as they can prevent women accessing postnatal care in low-income countries and the more positive consequences of having a 40-day period come from a ‘socially enforced’ rest and seclusion.

## Abbreviations

FCHV, female community health volunteers; FG, focus groups; I, interviews; MCHW, maternal and child health workers; PNC, postnatal care; WHO, World Health Organization
